# Post-match Recovery Practices in Professional Football: Design, Validity, and Reliability of a New Questionnaire

**DOI:** 10.3389/fspor.2021.680799

**Published:** 2021-07-15

**Authors:** Sérgio M. Querido, João Brito, Pedro Figueiredo, Filomena Carnide, João R. Vaz, Sandro R. Freitas

**Affiliations:** ^1^Faculty of Human Kinetics, Centro Interdisciplinar de Performance Humana, University of Lisboa, Lisbon, Portugal; ^2^Portugal Football School, Portuguese Football Federation, Oeiras, Portugal; ^3^Research Center in Sports Sciences, Health Sciences and Human Development, CIDESD, University Institute of Maia, ISMAI, Maia, Portugal

**Keywords:** fatigue, recovery assessment, survey, validation, soccer

## Abstract

**Introduction:** Although several approaches have been proposed to mitigate post-match fatigue, few studies have been conducted in team sports to understand the types of recovery methods and the underlying reasons for the choices of medical and technical staff. This study aimed to develop a valid and reliable online questionnaire to assess the recovery practices implemented by football clubs within 72 h post-match.

**Methods:** Two research members developed the original questionnaire proposal, and two experts in sports science and sports medicine confirmed the content and face validities. Then, 20 football coaches (age: 39.4 ± 6.8 years) with a minimum of 5 years of experience in professional football (9.1 ± 4.9 years) and with an academic background participated in determining the ecological validity and reliability of the questionnaire. The acceptability and relevance of the questionnaire were determined using descriptive statistics.

**Results:** After confirming the content and face validities, one questionnaire section with two questions was excluded due to lack of relevance, seven open-ended questions were removed due to the adherence of small participants (i.e., 45.4%), and one section was divided into three to facilitate clearness in reading. The remaining sections were considered acceptable and relevant (>94.1%). About 91.8% of nominal and ordinal items derived from the questionnaire questions showed good to very good reliability outcomes (average *k* classification: 0.73 ± 0.13; min–max: 0.22–1.00, *p* < 0.05; average *wk* classification: 0.82 ± 0.15; min–max: 0.22–1.00, *p* < 0.05).

**Conclusions:** This study provided a novel, valid, reliable, and easy-to-use tool to examine the post-match recovery practices in professional football contexts.

## Introduction

Optimal recovery is fundamental to avoid long-term fatigue and adverse consequences such as under-recovery, non-functional overreaching, or overtraining syndrome (Doeven et al., 2018; Kellmann et al., 2018). This is particularly important in professional sports contexts, where the density of competitions may be high. Several approaches have been proposed to mitigate the post-match effects on physical impairments and to increase recovery kinetics (Nédélec et al., 2013; Abaidia and Dupont, 2018; Altarriba-Bartes et al., 2020b). However, different recovery methods have distinct degrees of effectiveness. For instance, Nédélec et al. (2013); Machado et al. (2016), and Abaidia and Dupont (2018) reported that hydration, adequate nutrition, adequate sleep routines, and the use of cold water immersion at 9–10°C for 10–20 min allow a reduction in muscle soreness and accelerate the recovery process. These practices appeared to shorten the recovery time in terms of restoring the initial level of performance, resulting in early readiness. Despite the improvements shown in perceptual ratings after the use of cold modalities, limited evidence exists regarding cooling effects on any other objective parameter, such as lactate levels, CK levels, IL-6 levels, or muscle strength, during a 96-h recovery period (Torres et al., 2012; Hohenauer et al., 2015). Similarly, the evidence that supports the effectiveness of active recovery, stretching, compression garments, massage, and electrical stimulation in professional teams is scarce (Nédélec et al., 2013). This creates difficulties among professionals when selecting the best recovery approaches for athletes.

The recovery practices used by professional football teams have been scarcely studied (Nédélec et al., 2013; Altarriba-Bartes et al., 2020b). To our knowledge, only three studies have been conducted in team sports to understand the types of recovery strategies and the underlying reasons for the choices of medical and technical staff (Van Wyk and Lambert, 2009; Nédélec et al., 2013; Altarriba-Bartes et al., 2020a). Taking everything into account, studies provided insights into the usage of recovery methods in high-performance team sports but did not specify the periods in which they should be used after competitions. It is important to note that the choice of recovery methods may be sport-dependent due to the sports-specific physiological demands. Likewise, the choice of recovery methods may also depend on the institutional socioeconomic context (Hoffmann et al., 2002). Therefore, it is important to characterize the recovery modalities in different sports and in different countries. Moreover, three studies have investigated the perception of an athlete regarding recovery practices and the effectiveness of several recovery modalities commonly used in team sports (Venter, 2014; Crowther et al., 2017; Tavares et al., 2017). However, the questionnaire has been directed to athletes who did not provide information underlying the decision-making. In addition, the aforementioned studies provided a generic view of recovery practices adopted but not in specific moments such as after competitions where the physiological and psychological demands are higher as compared to the training contexts (Nédélec et al., 2012; Silva et al., 2018).

As the accuracy of the given information is highly dependent on the validity and reliability of the data collection instrument (Hopkins, 1998; Leppink and Pérez-Fuster, 2017), it is important to first determine how well the new tool measures the underlying construct. Hopkins (1998) emphasized the point that questionnaires *per se* are not reliable and that research instruments lacking reliability cannot measure any variable better than chance alone.

This study aimed to develop and validate an online questionnaire to assess the recovery practices implemented in elite football within 72 h post-match. A high level of agreement between raters and high reliability was expected to be observed so that confidence could be obtained to use the questionnaire in future studies.

## Methods

### Study Design

The research project was divided into three phases. In phase I, online questionnaire content was developed. In phase II, the content and ecological validities of the questionnaire were determined. Finally, in phase III, the reliability of the questionnaire was determined.

### Participants

Two sports science researchers, with at least 5 years of experience with recovery methods in elite football, built the first proposal of the questionnaire (phase I). Subsequently, two professional experts in sports science (Ph.D. in Sports Science) and sports medicine (specialization in Sports Medicine), both with more than 10 years of experience in practice and research on recovery methods in professional football, were invited to participate in the content validity and face validity procedure (phase II). In addition, 20 Portuguese football coaches (age: 39.38 ± 6.79 years) with a minimum of 5 years of experience in professional football (i.e., 9.07 ± 4.92 years) and at least a bachelor degree were invited to participate in the questionnaire ecological validity (i.e., acceptability and relevance) procedure (phase II). The sample size for validation of the pre-test questionnaire was chosen considering a sample of 15 to 20 participants, as previously recommended (Sheatsley, 1983; Vieira, 2009; Perneger et al., 2015). Regarding the recommendation, a sample of 20 professionals that is sufficient to detect at least the occurrence of one problem with a statistical power of 90% in a prevalence of the problem of 0.11 has been proposed (Perneger et al., 2015). The same coaches who participated in phase II were also invited to participate in the reliability procedure of the questionnaire (phase III). The participants were invited by personal contact and/or by email contact between April 2019 and July 2019. This study was approved by the local ethics committee (approval number: 10/2019), and the procedures were conducted according to the principles expressed in the Declaration of Helsinki. All participants gave their written informed consent to participate in this study.

### Procedures

**Figure 1:**
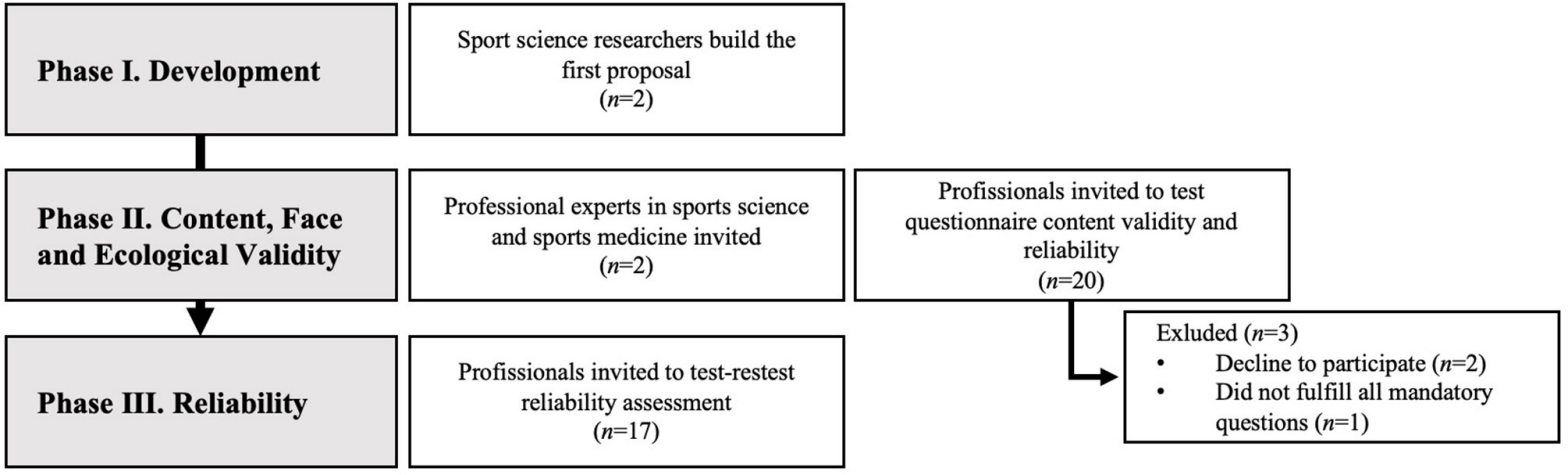
Flow diagram of sample attendance and questionnaire validation procedures.

This questionnaire was developed to be applied to the professionals responsible for the post-match recovery approaches in Portuguese professional football teams. It was assumed that these professionals hold an academic degree in the following: sports coaching, sports science, physical therapy, or sports medicine. The study phases and the sample attendance are shown in Figure 1.

In phase I (i.e., development), two researchers conceived the proposal of the original questionnaire based on the previous findings (Nédélec et al., 2013; Johnston et al., 2015; Owen et al., 2015). This procedure was used to define the construct of the questionnaire as no previous instrument has been conceived for a similar purpose. The questionnaire was written in Portuguese language and inserted in an online survey platform (LimeSurvey Open-Source platform, v3.17.9, LimeSurvey GmbH, Hamburg, Germany) to be filled online while assuring anonymity. In addition to the informed consent and personal information sections, questions were developed with the objective to (i) examine the importance given to the recovery intervention after the match and (ii) to characterize the type and extent of recovery method used at different post-match moments until 72 h after home and away matches. Closed-ended questions with nominal (variables with categories that do not have a natural order or ranking) and ordinal measurement scales (variables that have a natural order or ranking) and open-ended questions were considered. Closed-ended questions were designed through dichotomous and Likert scales with five categories.

In phase II, for the examination of content and face validities, two sports science and sports medicine experts commented on the initial proposal and proposed changes concerning whether the questionnaire contents were understandable and achieved the purpose of the questionnaire (Bolarinwa, 2015). Based on those comments, changes were made in the initial questionnaire by removing questions, changing their content, and altering the sequence of questions. To ensure the questionnaire was acceptable and relevant in an ecological setting (i.e., ecological validity), the invited professional football coaches completed the questionnaire and added some comments when justified. Based on those comments, changes were introduced in the questionnaire by removing questions that were not understandable and/or not considered relevant for the study purpose. Unanswered open-ended questions were removed (Vieira, 2009). The final version of the questionnaire was obtained at the end of phase II. After 7 days, the same professional football coaches who participated in ecological validation were asked to fulfill the questionnaire once again and, consequently, participated in test–retest questionnaire reliability (i.e., phase III).

### Data Analysis

Data analysis was performed using IBM SPSS Statistics (v26, IBM Corporation, New York, USA). Descriptive statistics were conducted to characterize the sample and to examine the acceptability and relevance of the questionnaire. Mean and SD were calculated for continuous variables, and absolute and relative frequencies were determined for nominal variables. Reliability testing was performed by calculating Cohen's kappa coefficient (*k*) and weighted Cohen's kappa coefficient (*wk*) for nominal and ordinal variables, respectively. The *k* and *wk* were classified as poor (<0.20), fair (0.20–0.39), moderate (0.40–0.59), good (0.60–0.79), and very good (0.80–1.00) (Landis et al., 1977).

## Results

A questionnaire with 34 questions, organized in five sections, was obtained in phase I (Supplementary File 1). The first two sections (i.e., five closed-ended questions and one open-ended question) were designed for informed consent and for the purpose of characterization of participants. Section Results (i.e., Portuguese language: reconhecimento da importância das práticas; English translation: recognition of the importance of practice) resulted in two closed-ended questions. Section Discussion (i.e., Portuguese language: caracterização das práticas; English translation: characterization of the practices) resulted in 11 closed-ended and 11 open-ended questions. Section Data Availability Statement (i.e., Portuguese language: o treino como estratégia preventiva; English translation: training as a preventive strategy) resulted in one closed-ended question and one open-ended question.

In phase II, based on the comments from experts, the questionnaire had the following changes: One section (with one closed-ended question and one open-ended question) was excluded due to lack of relevance; section Discussion (i.e., “caracterização das práticas”; characterization of the practices) was divided into three sections to facilitate the clearness in reading; and eight questions were modified to facilitate understanding. In addition, face validity was guaranteed for all items of the questionnaire. During the ecological validation (phase II) and reliability testing (phase III) processes, three participants were excluded (i.e., two did not accept the initial invitation, and one did not fulfill all mandatory questions); thus, only 17 participants accomplished all the steps. The acceptability and relevance outcomes are presented in Table 1.

**Table 1:** The number of positive responses among the participants (*n* = 17) concerning the acceptability, relevance, and suggestions of questionnaire sections during the ecological validation process.

**Questionnaire Section**	**Acceptability *n* (%)**	**Relevance *n* (%)**	**Suggestions *n* (%)**
1. *Consentimento* (Informed consent)	17 (100.0)	17 (100.0)	0 (0.0)
2. *Informações pessoais* (Personal details)	17 (100.0)	17 (100.0)	0 (0.0)
3. *Reconhecimento da importância das práticas* (Recognition of the importance of practices)	16 (94.1)	16 (94.1)	1 (5.9)
4. *Caracterização das práticas* (Characterization of the practices)	17 (100.0)	17 (100.0)	0 (0.0)

*n, number of responses*.

Questionnaire sections were considered acceptable and relevant by most of the participants (i.e., >94.1%). One participant did not accept and considered two questions of section Results as relevant (i.e., items C2 and C4) and suggested small changes related to the text format and instructions. The corrections suggested in the two items were implemented to facilitate the clearness in reading. In addition, seven open-ended questions were removed due to the adherence of small participants (i.e., 45.4%). A final questionnaire version comprising 19 questions separated into six sections was obtained (Supplementary File 2). From the reliability procedure of phase III, 91.8% of nominal and ordinal items derived from the questions of the questionnaire showed good to very good reliability outcomes (Table 2).

Table 2Reliability outcomes for nominal and ordinal items derived from the questions of the questionnaire.

**Table:** 

**Section 3**			**Section 4**			**Section 5**		
**Item**	**Cohen's *k* (k)**	**Classification**	**Item**	**Cohen's *k* (k)**	**Classification**	**Item**	**Cohen's *k* (k)**	**Classification**
**Nominal items**								
C2a	1.00	Very Good	D1	1.00	Very Good	D17	0.85	Very Good
C2b	0.70	Good	D3a	0.79	Good	D19	0.22	Fair
C2c	0.81	Very Good	D3b	0.71	Good			
C2d	0.66	Good	D3c	0.71	Good			
C2e	0.72	Good	D3d	0.64	Good			
C2f	0.55	Moderate	D3e	0.63	Good			
C2g	0.63	Good	D3f	0.89	Very Good			
C2h	0.35	Fair	D3g	0.82	Very Good			
C2i	1.00	Very Good	D3h	0.81	Very Good			
			D3i	1.00	Very Good			
			D8	0.71	Good

**Table:** 

**Section 3**			**Section 4**			**Section 5**		
**Item**	**Cohen's *k* (k)**	**Classification**	**Item**	**Cohen's *k* (k)**	**Classification**	**Item**	**Cohen's *k* (k)**	**Classification**
**Ordinal items**								
C1	0.60	Good	D6a	0.83	Very Good	D17	0.85	Very Good
C4a	0.70	Good	D6b	0.90	Very Good	D19	0.22	Fair
C4b	0,72	Good	D6c	0.92	Very Good	D15a	0.78	Good
C4c	0.70	Good	D6d	0.81	Very Good	D15b	0.81	Very Good
C4d	0.81	Very Good	D6e	0.78	Good	D15c	0.86	Very Good
C4e	0.84	Very Good	D6f	0.91	Very Good	D15d	0.85	Very Good
C4f	0.49	Good	D6g	0.57	Moderate	D15e	0.92	Very Good
C4g	0.22	Fair	D6h	0.46	Moderate	D15f	0.96	Very Good
C4h	1.0	Very Good	D6i	0.88	Very Good	D15g	0.87	Very Good
			D10a	0.83	Very Good	D15h	1.00	Very Good
			D10b	1.00	Very Good	D15i	0.84	Very Good
			D10c	0.94	Very Good	D21a	0.76	Good
			D10d	0.94	Very Good	D21b	1.00	Very Good
			D10e	0.90	Very Good	D21c	0.73	Good
			D10f	0.89	Very Good	D21d	0.75	Good
			D10g	0.89	Very Good	D21e	0.95	Very Good
			D10h	0.63	Good	D21f	0.95	Very Good
			D10i	1.00	Very Good	D21g	0.72	Good
			D12a	0.64	Good	D21h	0.63	Good
			D12b	0.92	Very Good			
			D12c	0.85	Very Good			
			D12d	0.77	Good			
			D12e	0.89	Very Good			
			D12f	0.89	Very Good			
			D12g	0.74	Good			
			D12h	0.81	Very Good			
			D12i	0.57	Moderate			

*k, Cohen's kappa coefficient; wk, weighted Cohen's kappa coefficient.*

For nominal items, first, in section Results (average *k* classification: 0.71 ± 0.21; range: 0.35–1.00, *p* < 0.01), one item showed a fair classification, one showed a moderate classification, four showed a good classification, and three showed a very good classification. Second, in section Discussion (average *k* classification: 0.79 ± 0.13; range: 0.63–1.00, *p* < 0.05), six items showed a good classification and five showed a very good classification. Finally, in section Data Availability Statement (average *k* classification: 0.54 ± 0.45; range: 0.22–1.00, *p* < 0.01), one item showed a fair classification, and one item showed a very good classification. For ordinal items, first, in section Results (average *wk* classification: 0.68 ± 0.22; range: 0.22–1.00, *p* < 0.01), one item showed a fair classification, five showed a good classification, and three showed a very good classification. Second, in section Discussion (average *k* classification: 0.82 ± 0.14; range: 0.46–1.00, *p* < 0.05), 3 items showed a moderate classification, 5 showed a good classification, and 19 showed a very good classification. Finally, in section Data Availability Statement (average *k* classification: 0.85 ± 0.11; range: 0.63–1.00, *p* < 0.01), 9 items showed a good classification and 18 items showed a very good classification.

## Discussion

This study aimed to develop a valid and reliable online questionnaire for the assessment of recovery practices implemented in elite football within 72 h post-match. A high level of agreement between raters and high reliability was obtained.

To our knowledge, only three studies have assessed recovery methods implemented by support sports staff in team sports through a questionnaire (Van Wyk and Lambert, 2009; Nédélec et al., 2013; Altarriba-Bartes et al., 2020a). Although good scientific contribution can be obtained from the aforementioned studies, we contend that some fundamental methodological aspects were disregarded. For instance, Van Wyk and Lambert (2009) determined the content validity by applying the proposed questionnaire in two moments for two different groups of individuals, which were reported to have similar characteristics to the target sample of the study. Although the characteristics were not mentioned, the comparison might have been affected, as individuals who evaluated the questionnaire were not the same. In contrast, Altarriba-Bartes et al. (2020a) ensured similar characteristics by applying a pilot test of the survey to two semiprofessional teams that were not included in the study. Similarly, Dadebo et al. (2004) conducted a survey to examine the relationship between stretching practices and hamstring injuries in English professional football. To ensure similar characteristics to the target sample, during the content validity process, the questionnaire was piloted by the responsible persons of three professional clubs, picked previously from the same study sample. In this study, content validity was confirmed doubly (i) by sports science/medicine experts and (ii) by football coaches with experience in the same football context and academic background. Moreover, first, in contrast to the previous studies, face validity was guaranteed by sports science/medicine experts following the procedures of Bolarinwa (2015).

Second, compliance with the questionnaire was not reported. In this study, acceptability and relevance were measured to ensure that the contents of the questionnaire were in line with the final purpose. Finally, the reliability of the questionnaires was not reported. As reliability reflects the repeatability of scores and the consistency over time (Hopkins, 1998; Leppink and Pérez-Fuster, 2017), it is very important to confirm where the instrument ensures a stable and representative response of participants over time. Thus, the present questionnaire provides accurate outcomes in terms of recovery practices in the post-match context of professional football.

In consequence of the demanding process of development, validation, and reliability processes, the present questionnaire presents some specific characteristics that define its context of use and increase the probability of its effectiveness. For instance, only closed-ended questions were included in the questionnaire, which is contrary to the studies proposed by Van Wyk and Lambert (2009) and Altarriba-Bartes et al. (2020a). On the one hand, open-ended questions allow responders to include more information about the subject, but, on the other hand, it can lead to a lot of noise that can make difficult the deep understanding behind the issue. We decided to remove open-ended questions due to the adherence of small participants, despite the known high reliability in these types of questions (Krosnick, 2018). In addition, questions with low response rates during a pre-test should be removed in the post-test (Vieira, 2009). In the same line, a response rate lower than 70% has been recommended as a cutoff to define whether questions should be removed (Dillman et al., 1974; Fan and Yan, 2010), which was adopted in this study. Additionally, based on the response of the participants during the validity process, the content of some questions was also modified in order to facilitate the clearness in reading. Section Discussion was also divided into three different sections based on the recommendations of Fan and Yan (2010), which reported that the layout design (i.e., screen-by-screen or scrolling layouts), text format for questions, and instructions significantly influenced the response rate. Moreover, in Likert-related questions, care was taken to have more than four options of response (Lozano et al., 2008) and with an odd number of options so that responders could choose a neutral response (Streiner et al., 2015). Thus, we have used questions with five options. We believe that all these procedures have contributed to the high reliability observed.

Despite the demanding validation process and high reliability observed, this study had some limitations. The exclusive use of a sample of Portuguese participants does not allow a generalization of the main findings to different contexts worldwide. The questionnaire criterion validity was also not assessed, as it would imply to assess the behaviors of coaches concerning the post-match recovery methods, which was not possible to implement. Additionally, a questionnaire provided in the Portuguese language does not allow further use in the context of different languages without having accomplished similar validity and reliability processes.

An important aspect that should be noted is the observed adhesion of high participants (85.0%). As indicated by Fan and Yan (2010), this may be because questionnaires promoted by academic and governmental agencies usually have higher response rates than those sponsored by commercial ones. Another factor that may contribute to the high adhesion is the intrinsic motivation of participants related to the subject of the questionnaire. Thus, future studies may consider the duration of experience and educational background of participants that may increase the predisposition to adhere to these types of assessments (i.e., questionnaires).

In conclusion, this study provides a novel, valid, reliable, and easy-to-use tool to examine post-match recovery practices in elite football contexts. Although the questionnaire was provided in Portuguese language, it can be used as a basis in other languages after its validation. From a practical perspective, besides the contribution of this tool in future research studies with the provision of accurate information, this study may also contribute to the knowledge of the current practices and methods in post-match recovery in professional football. In addition, this study may also help to clarify some divergences between theory (i.e., the effectiveness of methods) and practice (i.e., methods used) as shown in post-match recovery in professional football.

## Data Availability Statement

The original contributions generated for the study are included in the article/Supplementary Material, further inquiries can be directed to the corresponding author/s.

## Ethics Statement

The studies involving human participants were reviewed and approved by Ethics Committee of University of Lisbon, Faculty of Human Kinetics. The patients/participants provided their written informed consent to participate in this study.

## Author Contributions

SQ, JB, PF, JV, and SF: conceptualization. SQ, JB, and SF: acquisition of data. SQ, JB, PF, FC, JV, and SF: analysis and interpretation of data and review and editing. SQ: original drafting. JB and SF: supervision. All authors have read and agreed to the published version of the manuscript.

## Conflict of Interest

The authors declare that the research was conducted in the absence of any commercial or financial relationships that could be construed as a potential conflict of interest.
