# Supplementary material for: Post-match Recovery Practices in Professional Football: Design, Validity, and Reliability of a New Questionnaire
Source: Front Sports Act Living. 2021 Jul 15;3:680799. doi: 10.3389/fspor.2021.680799 (PMC8319234; doi:10.3389/fspor.2021.680799)
Supplement: Supplementary file 1 [file Data_Sheet_1.ZIP › file 1.pdf]

Secção A: Consentimento

A1. Aceito a participação e o preenchimento do questionário.

- Sim ☐
- Não ☐

Secção B: Detalhes Pessoais

B1. Qual o campeonato onde atua a equipa que representa?

- Primeira Liga ☐
- Segunda Liga ☐
- Liga Revelação ☐

B2. Qual a sua função no clube?

B3. Qual a sua formação académica?

- Ensino Básico ☐
- Ensino Secundário ☐
- Licenciatura ☐
- Mestrado ☐
- Doutoramento ☐
- Outro ☐

Outro

B4. Quanto tempo de experiência profissional tem no âmbito do treino desportivo de alto rendimento?

- Menos de 1 ano ☐
- 1 a 2 anos ☐
- 2 a 5 anos ☐
- 5 a 10 anos ☐
- Mais de 10 anos ☐

Secção C: Reconhecimento da Importância das Práticas

C1. Em quanto concorda com a seguinte expressão?

"As estratégias de recuperação são fundamentais para a recuperação dos atletas, até 72 horas, após o jogo."

Discordo Totalmente	Discordo	Neutro	Concordo	Concordo Totalmente
☐	☐	☐	☐	☐

C2. Seleccione e ordene as estratégias de recuperação pelo grau de importância que lhes atribui.

Coloque as estratégias de recuperação, que considera importantes, na coluna da direita e ordene-as pelo grau de importância (a estratégia que que considera mais importante deve estar em cima). Se não considerar outra estratégia de recuperação, poderá deixar o item na coluna da esquerda.

- Alongamento ☐
- Electro Estimulação ☐
- Recuperação Ativa ☐
- Imersão em Água Fria ☐
- Massagem ☐
- Meias e Calças de Compressão ☐
- Nutrição e Suplementação ☐
- Sono ☐
- Outra * ☐

C3. * No caso de ter considerado outra estratégia de recuperação, identifique qual.

C4. Qual a importância que atribui às seguintes estratégias de recuperação?

	Nada Importante	Pouco Importante	Importante	Muito Importante	Extremamente Importante
Alongamento	☐	☐	☐	☐	☐
Electro Estimulação	☐	☐	☐	☐	☐
Recuperação Ativa	☐	☐	☐	☐	☐
Imersão em Água Fria	☐	☐	☐	☐	☐
Massagem	☐	☐	☐	☐	☐

	Nada Importante	Pouco Importante	Importante	Muito Importante	Extremamente Importante
Meias e Calças de Compressão	☐	☐	☐	☐	☐
Nutrição e Suplementação	☐	☐	☐	☐	☐
Sono	☐	☐	☐	☐	☐
Outra *	☐	☐	☐	☐	☐

C5. *** No caso de ter considerado outra estratégia de recuperação, identifique qual.**

Secção D: Caracterização da Prática

D1. **Habitualmente, utilizam estratégias de recuperação?**

Sim ☐

Não ☐

D2. **Indique a(s) razão(ões) para não utilizar habitualmente estratégias de recuperação.**

D3. **Selecione e ordene as estratégias de recuperação pelo grau de utilização.**

Coloque as estratégias de recuperação que utiliza na coluna da direita e ordene-as pelo grau de utilização (a estratégia que mais utiliza deve estar em cima). A(s) estratégia(s) que não utiliza deixe na coluna da esquerda.

Alongamento ☐

Electro Estimulação ☐

Recuperação Ativa ☐

Imersão em Água Fria ☐

Massagem ☐

Meias e Calças de Compressão ☐

Nutrição e Suplementação ☐

Sono ☐

Outra * ☐

D4. *** No caso de considerar outra estratégia de recuperação, indique qual.**

D5. **Qual(ais) o(s) factor(es) que determina(m) a escolha e a aplicação das estratégias de recuperação?**

Por exemplo: Recursos disponíveis, perceção dos atletas, eficácia conhecida, etc.

D6. **Qual a frequência de utilização de métodos de recuperação, imediatamente após os jogos realizados em casa?**

	Nunca	Raramente	Às Vezes	Frequentemente	Sempre
Alongamento	☐	☐	☐	☐	☐
Electro Estimulação	☐	☐	☐	☐	☐
Recuperação Ativa	☐	☐	☐	☐	☐
Imersão em Água Fria	☐	☐	☐	☐	☐
Massagem	☐	☐	☐	☐	☐
Meias e Calças de Compressão	☐	☐	☐	☐	☐
Nutrição e Suplementação	☐	☐	☐	☐	☐
Sono	☐	☐	☐	☐	☐
Outra *	☐	☐	☐	☐	☐

D7. **No caso de ter considerado outra estratégia de recuperação, identifique qual.**

D8. **Nos jogos em casa, existe alguma consideração nas estratégias de recuperação quando o jogo é realizado de manhã, à tarde ou à noite?**

Sim ☐

Não ☐

D9. **Se sim, indique qual(ais).**

D10. **Qual a frequência de utilização de métodos de recuperação, 12 a 24 horas após os jogos realizados em casa?**

	Nunca	Raramente	Às Vezes	Frequentemente	Sempre
Alongamento	☐	☐	☐	☐	☐
Electro Estimulação	☐	☐	☐	☐	☐
Recuperação Ativa	☐	☐	☐	☐	☐
Imersão em Água Fria	☐	☐	☐	☐	☐
Massagem	☐	☐	☐	☐	☐
Meias e Calças de Compressão	☐	☐	☐	☐	☐
Nutrição e Suplementação	☐	☐	☐	☐	☐
Sono	☐	☐	☐	☐	☐
Outra *	☐	☐	☐	☐	☐

D11. *** No caso de ter considerado outra estratégia de recuperação, identifique qual.**

D12. **Qual a frequência de utilização de métodos de recuperação, 24 a 72 horas após os jogos realizados em casa?**

	Nunca	Raramente	Às Vezes	Frequentemente	Sempre
Alongamento	☐	☐	☐	☐	☐
Electro Estimulação	☐	☐	☐	☐	☐
Recuperação Ativa	☐	☐	☐	☐	☐
Imersão em Água Fria	☐	☐	☐	☐	☐
Massagem	☐	☐	☐	☐	☐
Meias e Calças de Compressão	☐	☐	☐	☐	☐

	Nunca	Raramente	Às Vezes	Frequentemente	Sempre
Nutrição e Suplementação	☐	☐	☐	☐	☐
Sono	☐	☐	☐	☐	☐
Outra *	☐	☐	☐	☐	☐

D13. *** No caso de ter considerado outra estratégia de recuperação, identifique qual.**

D14. **Qual a periodização, dosagem e modos de aplicação das estratégias que usa?**

D15. **Qual a frequência de utilização de métodos de recuperação, imediatamente após os jogos realizados fora?**

	Nunca	Raramente	Às Vezes	Frequentemente	Sempre
Alongamento	☐	☐	☐	☐	☐
Electro Estimulação	☐	☐	☐	☐	☐
Recuperação Activa	☐	☐	☐	☐	☐
Imersão em Água Fria	☐	☐	☐	☐	☐
Massagem	☐	☐	☐	☐	☐
Meias e Calças de Compressão	☐	☐	☐	☐	☐
Nutrição e Suplementação	☐	☐	☐	☐	☐
Sono	☐	☐	☐	☐	☐
Outra *	☐	☐	☐	☐	☐

D16. *** No caso de ter considerado outra estratégia de recuperação, identifique qual.**

D17. **Nos jogos fora, existe alguma consideração nas estratégias de recuperação quando o jogo é realizado de manhã, à tarde ou à noite?**

- [ ] Sim
- [ ] Não

D18. **Se sim, indique qual(ais).**

D19. **Nos jogos fora, existe alguma consideração nas estratégias de recuperação quando a viagem de regresso é superior a 4 horas?**

- [ ] Sim
- [ ] Não

D20. **Se sim, indique qual(ais).**

D21. **Qual a frequência de utilização de métodos de recuperação, 12 a 24 horas após os jogos realizados fora?**

	Nunca	Raramente	Às Vezes	Frequentem ente	Sempre
Alongamento	☐	☐	☐	☐	☐
Electro Estimulação	☐	☐	☐	☐	☐
Recuperação Ativa	☐	☐	☐	☐	☐
Imersão em Água Fria	☐	☐	☐	☐	☐
Massagem	☐	☐	☐	☐	☐
Meias e Calças de Compressão	☐	☐	☐	☐	☐
Nutrição e Suplementação	☐	☐	☐	☐	☐
Sono	☐	☐	☐	☐	☐
Outra *	☐	☐	☐	☐	☐

D22. *** No caso de ter considerado outra estratégia de recuperação, identifique qual.**

D23. **Qual a frequência de utilização de métodos de recuperação, 24 a 72 horas após os jogos realizados fora?**

	Nunca	Raramente	Às Vezes	Frequentemente	Sempre
Alongamento	☐	☐	☐	☐	☐
Electro Estimulação	☐	☐	☐	☐	☐
Recuperação Ativa	☐	☐	☐	☐	☐
Imersão em Água Fria	☐	☐	☐	☐	☐
Massagem	☐	☐	☐	☐	☐
Meias e Calças de Compressão	☐	☐	☐	☐	☐
Nutrição e Suplementação	☐	☐	☐	☐	☐
Sono	☐	☐	☐	☐	☐
Outra	☐	☐	☐	☐	☐

D24. *** No caso de ter considerado outra estratégia de recuperação, identifique qual.**

D25. **Qual a periodização, dosagem e modos de aplicação das estratégias que usa?**

Secção E: O Treino Como Estratégia Preventiva

E1. **Existe alguma preocupação em utilizar o treino da força e da velocidade como estratégia preventiva para reduzir o dano muscular induzido pelo jogo?**

Sim ☐

Não ☐

E2. Se sim, explique de que forma é realizado o planeamento.
