# Supplementary material for: Post-match Recovery Practices in Professional Football: Design, Validity, and Reliability of a New Questionnaire
Source: Front Sports Act Living. 2021 Jul 15;3:680799. doi: 10.3389/fspor.2021.680799 (PMC8319234; doi:10.3389/fspor.2021.680799)
Supplement: Supplementary file 1 [file Data_Sheet_1.ZIP › file 2.pdf]

Secção A: Consentimento

A1. **Aceito a participação e o preenchimento do questionário.**

- Sim ☐
- Não ☐

Secção B: Detalhes Pessoais

B1. **Qual o campeonato onde atua a equipa que representa?**

- Primeira Liga ☐
- Segunda Liga ☐
- Liga Revelação ☐
- Primeira Divisão Feminina ☐
- Outro ☐

Outro

B2. **Qual a sua função no clube?**

B3. **Qual a sua formação académica?**

- Ensino Básico ☐
- Ensino Secundário ☐
- Licenciatura ☐
- Mestrado ☐
- Doutoramento ☐
- Outro ☐

Outro

B4. **Quanto tempo de experiência profissional tem no âmbito do treino desportivo de alto rendimento?**

- Menos de 1 ano ☐
- 1 a 2 anos ☐
- 2 a 5 anos ☐
- 5 a 10 anos ☐
- Mais de 10 anos ☐

Secção C: Reconhecimento da Importância das Práticas

C1. **Em quanto concorda com a seguinte expressão?**

"As estratégias de recuperação são fundamentais para a recuperação dos atletas, até 72 horas, após o jogo."

Discordo Totalmente	Discordo	Neutro	Concordo	Concordo Totalmente
☐	☐	☐	☐	☐

C2. **Seleccione e ordene as seguintes estratégias de recuperação pela importância que lhes atribui.**

Coloque as estratégias de recuperação, que considera importantes, na coluna da direita e ordene-as pelo grau de importância (a estratégia que que considera mais importante deve estar em cima). Se não considerar outra estratégia de recuperação, poderá deixar o item na coluna da esquerda.

- Alongamento ☐
- Electro Estimulação ☐
- Recuperação Ativa ☐
- Imersão em Água Fria ☐
- Massagem ☐
- Meias e Calças de Compressão ☐
- Nutrição e Suplementação ☐
- Sono ☐
- Outra * ☐

C3. *** No caso de ter considerado outra estratégia de recuperação, identifique qual.**

C4. Qual o grau de importância que atribui às seguintes estratégias de recuperação?

	Nada Importante	Pouco Importante	Importante	Muito Importante	Extremamente Importante
Alongamento	☐	☐	☐	☐	☐
Electro Estimulação	☐	☐	☐	☐	☐
Recuperação Ativa	☐	☐	☐	☐	☐
Imersão em Água Fria	☐	☐	☐	☐	☐
Massagem	☐	☐	☐	☐	☐
Meias e Calças de Compressão	☐	☐	☐	☐	☐
Nutrição e Suplementação	☐	☐	☐	☐	☐
Sono	☐	☐	☐	☐	☐

Secção D: Caracterização da Prática

D1. Habitualmente, utilizam estratégias de recuperação?

Sim ☐

Não ☐

D2. Selecione e ordene as seguintes estratégias de recuperação em função da frequência de utilização.

Coloque as estratégias de recuperação que utiliza na coluna da direita e ordene-as pelo grau de utilização (a estratégia que mais utiliza deve estar em cima). A(s) estratégia(s) que não utiliza deixe na coluna da esquerda.

Alongamento ☐

Electro Estimulação ☐

Recuperação Ativa ☐

Imersão em Água Fria ☐

Massagem ☐

Meias e Calças de Compressão ☐

Nutrição e Suplementação ☐

Sono ☐

Outra * ☐

D3. *** No caso de considerar outra estratégia de recuperação, indique qual.**

Secção E: Caracterização da Prática nos Jogos em Casa

E1. **Imediatamente após os jogos realizados em casa, qual a frequência de utilização das seguintes estratégias de recuperação?**

	Nunca	Raramente	Às Vezes	Frequentemente	Sempre
Alongamento	☐	☐	☐	☐	☐
Electro Estimulação	☐	☐	☐	☐	☐
Recuperação Ativa	☐	☐	☐	☐	☐
Imersão em Água Fria	☐	☐	☐	☐	☐
Massagem	☐	☐	☐	☐	☐
Meias e Calças de Compressão	☐	☐	☐	☐	☐
Nutrição e Suplementação	☐	☐	☐	☐	☐
Sono	☐	☐	☐	☐	☐
Outra *	☐	☐	☐	☐	☐

E2. **No caso de ter considerado outra estratégia de recuperação, identifique qual.**

E3. **Nos jogos realizados em casa, existe alguma consideração nas estratégias de recuperação quando o jogo é realizado de manhã, à tarde ou à noite?**

Sim ☐

Não ☐

E4. **12 a 24 horas após os jogos realizados em casa, qual a frequência de utilização das seguintes estratégias de recuperação?**

	Nunca	Raramente	Às Vezes	Frequentemente	Sempre
Alongamento	☐	☐	☐	☐	☐
Electro Estimulação	☐	☐	☐	☐	☐
Recuperação Ativa	☐	☐	☐	☐	☐
Imersão em Água Fria	☐	☐	☐	☐	☐
Massagem	☐	☐	☐	☐	☐
Meias e Calças de Compressão	☐	☐	☐	☐	☐
Nutrição e Suplementação	☐	☐	☐	☐	☐
Sono	☐	☐	☐	☐	☐
Outra *	☐	☐	☐	☐	☐

E5. *** No caso de ter considerado outra estratégia de recuperação, identifique qual.**

E6. **24 a 72 horas após os jogos realizados em casa, qual a frequência de utilização das seguintes estratégias de recuperação?**

	Nunca	Raramente	Às Vezes	Frequentemente	Sempre
Alongamento	☐	☐	☐	☐	☐
Electro Estimulação	☐	☐	☐	☐	☐
Recuperação Ativa	☐	☐	☐	☐	☐
Imersão em Água Fria	☐	☐	☐	☐	☐
Massagem	☐	☐	☐	☐	☐
Meias e Calças de Compressão	☐	☐	☐	☐	☐
Nutrição e Suplementação	☐	☐	☐	☐	☐
Sono	☐	☐	☐	☐	☐
Outra *	☐	☐	☐	☐	☐

E7. *** No caso de ter considerado outra estratégia de recuperação, identifique qual.**

Secção F: Caracterização da Prática nos Jogos Fora

F1. **Imediatamente após os jogos realizados fora, qual a frequência de utilização das seguintes estratégias de recuperação?**

	Nunca	Raramente	Às Vezes	Frequentemente	Sempre
Alongamento	☐	☐	☐	☐	☐
Electro Estimulação	☐	☐	☐	☐	☐
Recuperação Activa	☐	☐	☐	☐	☐
Imersão em Água Fria	☐	☐	☐	☐	☐
Massagem	☐	☐	☐	☐	☐
Meias e Calças de Compressão	☐	☐	☐	☐	☐
Nutrição e Suplementação	☐	☐	☐	☐	☐
Sono	☐	☐	☐	☐	☐
Outra *	☐	☐	☐	☐	☐

F2. *** No caso de ter considerado outra estratégia de recuperação, identifique qual.**

F3. **Nos jogos realizados fora, existe alguma consideração nas estratégias de recuperação quando o jogo é realizado de manhã, à tarde ou à noite?**

Sim ☐

Não ☐

F4. **Nos jogos realizados fora, existe alguma consideração nas estratégias de recuperação quando a viagem de regresso é superior a 4 horas?**

Sim ☐

Não ☐

F5. **12 a 24 horas após os jogos realizados fora, qual a frequência de utilização das seguintes estratégias de recuperação?**

	Nunca	Raramente	Às Vezes	Frequentemente	Sempre
Alongamento	☐	☐	☐	☐	☐
Electro Estimulação	☐	☐	☐	☐	☐
Recuperação Ativa	☐	☐	☐	☐	☐
Imersão em Água Fria	☐	☐	☐	☐	☐
Massagem	☐	☐	☐	☐	☐
Meias e Calças de Compressão	☐	☐	☐	☐	☐
Nutrição e Suplementação	☐	☐	☐	☐	☐
Sono	☐	☐	☐	☐	☐
Outra *	☐	☐	☐	☐	☐

F6. *** No caso de ter considerado outra estratégia de recuperação, identifique qual.**

F7. **24 a 72 horas após os jogos realizados fora, qual a frequência de utilização das seguintes estratégias de recuperação?**

	Nunca	Raramente	Às Vezes	Frequentemente	Sempre
Alongamento	☐	☐	☐	☐	☐
Electro Estimulação	☐	☐	☐	☐	☐
Recuperação Ativa	☐	☐	☐	☐	☐
Imersão em Água Fria	☐	☐	☐	☐	☐
Massagem	☐	☐	☐	☐	☐
Meias e Calças de Compressão	☐	☐	☐	☐	☐
Nutrição e Suplementação	☐	☐	☐	☐	☐
Sono	☐	☐	☐	☐	☐
Outra	☐	☐	☐	☐	☐

F8. *** No caso de ter considerado outra estratégia de recuperação, identifique qual.**
